# Supplementary material for: TARGETing secondary school students’ motivation towards physical education: The role of student-perceived mastery climate teaching strategies
Source: PLoS One. 2022 Sep 22;17(9):e0274964. doi: 10.1371/journal.pone.0274964 (PMC9499303; doi:10.1371/journal.pone.0274964)
Supplement: S1 Questionnaire — (DOCX) [file pone.0274964.s002.docx]

# **S1 Questionnaire. Mastery Teaching Perception Questionnaire (MTP-Q).**

*Introduction*: Please think about your experiences within PE throughout this school year. What was it usually like? Read the following statements carefully and respond to each statement based on your experiences. How students perceive PE class naturally vary from student to student, so be certain to take your time and answer as honestly as possible. Pick the number that best represents your experiences.

*Note*: Each item is responded to on a 5-point Likert-type scale (1 = strongly disagree; 5 = strongly agree).

| **Nr.** | **Item** |
| --- | --- |
| 1 | In general, there is plenty of variety and alternation in the PE lessons. |
| 2 | Our PE teacher provides me with opportunities to deliver input and ideas during PE lessons (e.g., by proposing changes to improve lesson activities). |
| 3 | During PE lessons students are primarily assessed on personal growth and development, not on the end result or the final performance. |
| 4 | Student groups are regularly reshuffled, so that students get to work with different peers. |
| 5 | Our PE teacher emphasizes mainly our successes. If students cannot perform the given task successfully yet, our PE teacher encourages us and provides students with useful feedback. |
| 6 | At the beginning of a task/activity and after a brief instruction, our PE teacher allows the students to start with the task/activity very quickly. |
| 7 | As students, we are actively involved in evaluation/assessment activities (e.g., by working with observation sheets or video-recordings that help us provide each other with feedback for further improvement). |
| 8 | It doesn’t matter what your skill level in PE is, our PE teacher finds it more important for students to show effort and involvement during PE lessons. |
| 9 | During the PE lessons our PE teacher constantly monitors if the student groups are fair and functional, and adjust them if needed. |
| 10 | From the outset we as students are well informed by our PE teacher about the learning goals and assessment criteria, what is expected from us, and what is needed to be done to succeed. |
| 11 | During PE lessons our PE teacher provides the students with truly challenging and attractive tasks/activities. |
| 12 | Our PE teacher really takes the students seriously and expects us to take a certain responsibility during PE lessons. |
| 13 | Our PE teacher provides me with a lot of feedback and clarifies how I can improve (even) further during the task/activity. |
| 14 | During the grouping process our PE teacher ensures that all students feel equally valued and that no-one is excluded. |
| 15 | When I am assessed, the assessment produces a reliable and honest representation of my capabilities. |
| 16 | All tasks/activities during PE are achievable for me. My capabilities are taken into account. |
| 17 | As students we are regularly provided with choice during tasks/activities and within the PE lessons in general. |
| 18 | Our PE teacher is closely involved in the PE lessons, paying attention to all students and is really interested in students. |
| 19 | When working in student groups, all students (e.g., boys and girls, skilled and less-skilled, competitive and less-competitive) are distributed well, so that we can learn from each other. |
| 20 | During assessment within PE, student differences are taken into account (e.g., height, weight, strength, agility, speed, motor skills). |
| 21 | Students hardly ever stand/sit around waiting during tasks/activities. All students are provided with sufficient opportunities to be physically active and actively participate within PE lessons. |
| 22 | Our PE teacher allow students to think along and assist in the grouping process. |
| 23 | Our PE teacher provides me with personal attention and feedback as much as possible. |
| 24 | During competitive play our PE teacher ensures balanced student teams which enables to play fair games. |
| 25 | During the learning process I am provided with sufficient information about my current performance level and how to do better in order to succeed during the assessment. |
| 26 | During tasks/activities in PE, it is always very clear to me what we need to do and what the (playing)rules are. |

| **Construct** | **Items** | **Item total** | **Cronbach’s alpha** |
| --- | --- | --- | --- |
| TASK | 1 + 6 + 11 + 16 + 21 + 26 | 6 | .79 |
| AUTHORITY | 2 + 7 + 12 + 17 + 22 | 5 | .79 |
| RECOGNITION | 5 + 8 + 13 + 18 + 23 | 5 | .85 |
| GROUPING | 4 + 9 + 14 + 19 + 24 | 5 | .80 |
| EVALUATION | 3 + 10 + 15 + 20 + 25 | 5 | .82 |
