# Supplementary material for: TARGETing secondary school students’ motivation towards physical education: The role of student-perceived mastery climate teaching strategies
Source: PLoS One. 2022 Sep 22;17(9):e0274964. doi: 10.1371/journal.pone.0274964 (PMC9499303; doi:10.1371/journal.pone.0274964)
Supplement: Attachment — Submitted filename: Response to reviewers.docx [file pone.0274964.s003.docx]

PONE-D-22-02985

TARGETing secondary school students’ motivation towards physical education: The role of student-perceived mastery climate teaching strategies.

PLOS ONE

Dear dr. Francisco Javier Huertas-Delgado,

Thank you for the opportunity to revise and resubmit our manuscript. Below, we will address all comments made by you and the reviewers.

**Editor’s comments:**

1. Please ensure that your manuscript meets PLOS ONE's style requirements, including those for file naming. We have changed the file names accordingly and have attempted to meet all PLOS ONE style requirements. This included, for example, changing header font sizes, adapting table headers and changing the in-text references from round to square brackets.

2. We note that you have included the phrase “data not shown” in your manuscript. Unfortunately, this does not meet our data sharing requirements. PLOS does not permit references to inaccessible data. We require that authors provide all relevant data within the paper, Supporting Information files, or in an acceptable, public repository. Please add a citation to support this phrase or upload the data that corresponds with these findings to a stable repository (such as Figshare or Dryad) and provide and URLs, DOIs, or accession numbers that may be used to access these data. Or, if the data are not a core part of the research being presented in your study, we ask that you remove the phrase that refers to these data.

We have removed the phrase ‘data not shown’. We meant to indicate that the data was only shown in-text, not in a table.

3. Please include captions for your Supporting Information files at the end of your manuscript, and update any in-text citations to match accordingly. Please see our Supporting Information guidelines for more information: <http://journals.plos.org/plosone/s/supporting-information>.

We have changed this accordingly.

4. Please review your reference list to ensure that it is complete and correct. If you have cited papers that have been retracted, please include the rationale for doing so in the manuscript text, or remove these references and replace them with relevant current references. Any changes to the reference list should be mentioned in the rebuttal letter that accompanies your revised manuscript. If you need to cite a retracted article, indicate the article’s retracted status in the References list and also include a citation and full reference for the retraction notice.

We have thoroughly reformatted all references, including addition of doi’s to all papers. As far as we are aware, there is now no citation of retracted papers.

**Reviewers' comments:**

Reviewer #1: 

Introduction:
It is a manuscript, not a chapter, so I recommend to reduce and summarize the information of this section. It is important to note the significant number of references incorporated in the introduction, although they are not very recent. The structure of the introduction follows the submission guidelines. The aim of the research is clear and relevant.

We have abbreviated this section by removing some of the more ‘textbook’-like information, e.g. elaborate information about SDT-motivational regulations, as well as the practical examples of lessons strategies associated with TARGET structures. We agree that these do not necessarily add to the understanding of our research objectives.

Also, we have deleted a number of references that were basically ‘overlapping’, making sure to retain the more recent ones where possible (see later).

Methods:
The research design seems to fit the purpose of the research. The instruments used for data collection are well described.

Results
The results section is well structured and very detailed.

Discussion and conclusions:
It is important to note the significant number of references incorporated in the discussion, although they are not very recent.
The conclusions are presented clearly and are justified by the results presented.

References:
Barely 25% of the references are later than 2016. It is necessary to include more up-to-date bibliography. If it is a topic of interest, it should be evidenced in the knowledge generated in recent years or, at least, in the discrepancies and suggestions of other authors, on the importance of addressing research on this topic.\

We have added a number of more recent references, so that now 49% of all references is post-2016.

The format of references should be revised to comply with the submission guidelines (<https://journals.plos.org/plosone/s/submission-guidelines>).

We have changed the format of the references to comply with the journal’s guidelines.

Reviewer #2: The manuscript is extremely interesting and well written. However, it would be necessary some reviews in the manuscript before been publish. There are two topics that need to be clear informed.

[page 8, line 165; 171-174] Ethics statement should be written in a specific topic in methods section. And more details about the institution where the project was approved.
We have added more info about the ethical procedures and approval and combined this into one paragraph (183-188).

[page 8, line 167] Why a convenience sample was selected instead of a probabilistic sample? Wasn’t this an additional limitation of the study? Also, it was not clear the eligibility criteria for specialist PE teachers being selected for the study. Would be interesting for readers to have some information about the universe population of specialist PE teachers in Netherlands.

We added text to the limitation to acknowledge this (501-505). Also, we included some information about (specialist) PE teachers in secondary education in the Netherlands (192-195).
